# Blood on the Medical Moon

**Published:** 1893-07

**Authors:** 


BLOOD ON THE MEDICAL MOON.—Culbertson, so long known as the fighting editor of the *Cincinnati Lancet Clinic*, was not a candidate for re-election to the editorial chair of the *Journal of the American Medical Association* at the recent meeting (a Doctor Crandall, of New York, was elected his successor). In the June 24th issue of the Journal, Culbertson has his validictory editorial, in which he reviews the Journal's business under his two year editorial management, and winds up with a red hot denunciations of one Dr. R. Harvey Reed, whom he accuses of having issued a type-writtten circular, stating that under Culbertson the Journal had gotten $10,000 in debt. This statement Dr. C. denounces as outrageously false and in law, libelous, and the action of the writer as infamous, and says, "To be a fool and a crank does not excuse a man for a crime." It strikes us Reed has got to fight. Culbertson is back at the helm of his splendid little weekly, the *Lancet Clinic*, which he should never have left.

